# A meta-analysis of the efficacy of allopurinol in reducing the incidence of myocardial infarction following coronary artery bypass grafting

**DOI:** 10.1186/s12872-018-0881-6

**Published:** 2018-07-11

**Authors:** Tejas P. Singh, Tristan Skalina, Daniel Nour, Aarya Murali, Sean Morrison, Joseph V. Moxon, Jonathan Golledge

**Affiliations:** 1 Queensland Research Centre for Peripheral Vascular Disease, College of Medicine and Dentistry, James Cook University, Townsville, QLD 4811 Australia; 2 The Department of Vascular and Endovascular Surgery, The Townsville Hospital, Townsville, QLD Australia

**Keywords:** Myocardial infarction, Allopurinol, Atherosclerosis

## Abstract

**Background:**

The xanthine oxidase inhibitor allopurinol that is commonly used to treat gout, has been suggested to have pleiotropic effects that are likely to reduce the incidence of myocardial infarction (MI) in at risk individuals. The aim of this meta-analysis was to assess the efficacy of allopurinol treatment in reducing the incidence of MI.

**Method:**

MEDLINE, Scopus, Web of Science, and Cochrane Library databases were searched for randomised controlled trials examining the efficacy of allopurinol in reducing the incidence of MI. The quality of study methodology was assessed by two independent reviewers using the Cochrane Collaboration’s tool for assessing risk of bias. This meta-analysis was conducted using a fixed-effects model, and heterogeneity was assessed with the I^2^ index.

**Results:**

One thousand one hundred twenty-three citations were screened and only six studies satisfied the inclusion criterion. Published between 1988 and 1995, all studies examined the cardioprotective efficacy of allopurinol in the setting of coronary artery bypass graft (CABG). From a total pooled sample size of 229, MI was reported in 2 (1.77%) allopurinol and 14 (12.07%) control patients. A fixed-effects meta-analysis (I^2^ = 0%) identified a statistically significant reduced incidence of myocardial infarction (RR 0.21, 95% CI: 0.06, 0.70, *p* = 0.01) in patients allocated to allopurinol. However, in the leave-one-out sensitivity analyses, the treatment effect became non-significant with the removal of one of the studies.

**Conclusion:**

Based on the limited evidence available, allopurinol appears to reduce the incidence of perioperative MI following CABG. Further research is required to confirm these findings.

**Electronic supplementary material:**

The online version of this article (10.1186/s12872-018-0881-6) contains supplementary material, which is available to authorized users.

## Background

Myocardial infarction (MI) is a common and frequently fatal endpoint of ischaemic heart disease. The World Health Organization estimates that, every year, MI accounts for 7.3 million deaths worldwide [1]. Among survivors, MI contributes to significant long-term morbidity due to complications such as congestive cardiac failure and increased risks of recurrent MI, stroke and death. Allopurinol is a xanthine oxidase inhibitor (XOI) that is widely prescribed for the treatment of gout due to its action to lower uric acid levels. There is also growing interest in a possible role of allopurinol in preventing and treating cardiovascular disease [2–4]. Allopurinol has been suggested to provide cardiovascular benefits as a result of three key actions: (1) reducing serum concentrations of uric acid which has pro-inflammatory effects [2, 5, 6]; (2) inhibiting xanthine oxidase mediated generation of reactive oxygen species which promote endothelial dysfunction and atherosclerosis plaque instability [2, 3, 7–12]*;* and (3) inhibiting purine catabolism thereby increasing local tissue availability of adenosine triphosphate and oxygen [8, 13, 14]. Through these actions allopurinol has the potential to limit atherosclerosis, prevent acute ischaemic events and protect against ischaemia-reperfusion injury. Previous studies investigating the efficacy of allopurinol as a cardiovascular drug have led to conflicting results [3, 5, 15]. Several large human association studies have linked the long-term use of allopurinol to a decreased risk of first-ever MI, recurrent MI and non-fatal MI [16–21]. In contrast, two small randomised trials of patients with cardiac failure reported no clinical benefits of allopurinol or its active metabolite, oxypurinol [22, 23]. No previous meta-analysis has examined the efficacy of allopurinol in the prevention of MI. The aim of this study was to perform a systematic review and meta-analysis of randomised controlled trials which have examined the efficacy of allopurinol in reducing the incidence of MI.

## Methods

This systematic review and meta-analysis was performed in accordance with the Preferred Reporting Items of Systematic Reviews and Meta-Analyses (PRISMA) statement [24]. A comprehensive search was conducted to identify all randomised controlled trials investigating the influence of allopurinol on the incidence of MI. MEDLINE (1966), Scopus (1996), Web of Science (1965), and Cochrane Library databases (1992) were searched from inception to 1st of June 2017.The following terms were used in keyword/topic searches across all databases: [allopurinol OR XOI* OR “xanthine oxidase inhibit*”] AND [“myocardial infarct*” OR MI OR “heart attack*” OR acute MI OR “acute coronary syndrome*” OR ACS OR “myocardial ischemia” OR “myocardial ischaemia” OR “myocardial necrosis” OR STEMI OR STEACS OR NSTEMI OR NSTEACS]. No limits were applied.

Citations from all four databases were pooled and screened by four independent reviewers (A.M., S.M, D.N. and T.S). After removal of duplicate results, titles and abstracts were screened to identify studies eligible for full text review. In the event of any uncertainty, full texts were evaluated. For inclusion into this review, the following criterion were required to be met: (1) original research publication; (2) randomised controlled trial design; (3) clear comparison between patient groups on allopurinol and control groups receiving no treatment/placebo; and (4) assessment and publication of data specific to incidence of MI, in allopurinol and control groups. Studies were excluded if no English language full text was available. Reference lists of included studies were hand-searched to increase the yield of relevant studies.

Data extraction was performed by two independent reviewers (A.M and S.M.) using detailed predefined forms endorsed by the Cochrane Collaboration and tailored to the requirements of this review. Salient information on study design, allopurinol/control treatment protocol, confounding factors, MI diagnostic criterion and MI incidence were extracted. A consensus meeting was held to critically discuss the extracted data and resolve inconsistencies between reviewers.

Study quality was assessed by two independent reviewers (A.M. and S.M.) using The Cochrane Collaboration’s tool for assessing risk of bias as published in the Cochrane Handbook 5.1.0 in 2011 [25]. Randomisation, allocation concealment, blinding and outcome reporting were critically evaluated. The risks of selection bias, performance bias, detection bias, attrition bias and reporting bias were judged to be high, low or unclear based on the rubric provided in the Cochrane Handbook 5.1.0. RevMan 5.3 was used to generate a summary table comparing the risks of bias within and between included studies.

All numeric data was entered into Microsoft Excel to perform basic quantitative analyses. Summary statistics including percentage values and measures of central tendency were obtained. A meta-analysis was performed to quantify the effect of allopurinol in preventing MI. For each study and each patient cohort i.e. allopurinol and control cohorts, sample sizes and absolute numbers of MI were recorded and analysed in RevMan 5.3. The I^2^ statistic was used to assess statistical heterogeneity between the included studies. A value greater than 50% was considered to represent substantial heterogeneity as previously described [25]. Due to the small sample sizes, low event numbers and minimal statistical heterogeneity among all included studies, Mantel-Haenszel methods were used to generate a fixed-effects model [26].

All effect sizes were reported using risk ratios (RR) with 95% confidence intervals (CI). *P*-values < 0.05 were considered statistically significant. Results from these analyses were graphically presented in forest plots. Assessment of publication bias using funnel plots was planned if there were a sufficient number of studies (*N* ≥ 10) [27].

## Results

**Fig. 1:**
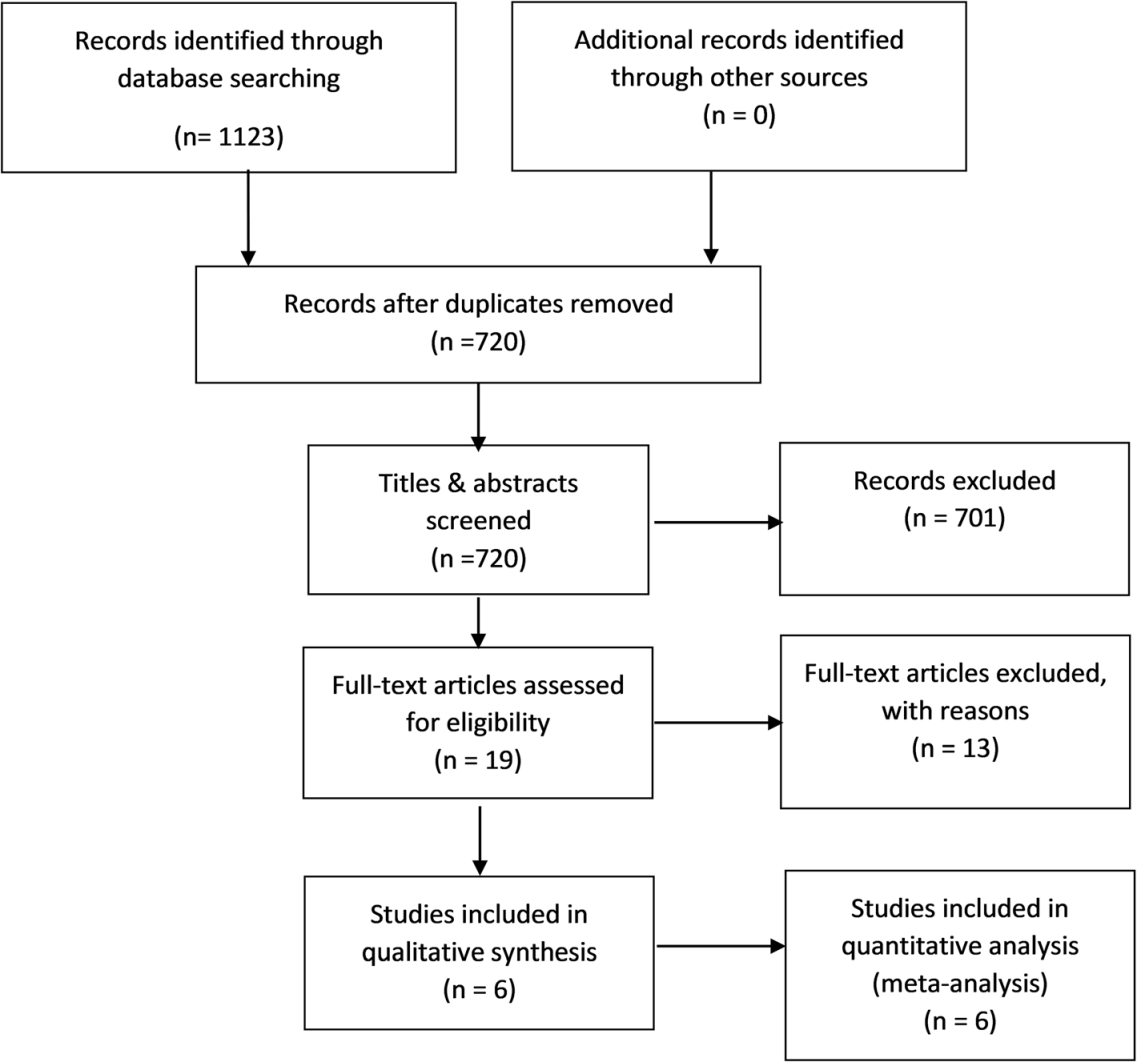
Outline of the study selection for this systematic review and meta-analysis

Initial searches yielded a total of 1123 citations. After removing duplicates, 720 articles were screened for eligibility based on review of their titles and abstracts. Seven hundred and one articles were excluded and 19 full texts were chosen for full text review (Fig. 1). The most common reasons for exclusion were: (1) not a randomised controlled trial; (2) absence of a clear comparison between a patient group receiving allopurinol and a control group receiving no treatment or placebo; and (3) absence of MI as an end-point that was reported [28]. Overall, 6 studies were chosen for inclusion into qualitative analysis [15, 29–33], of which 4 were specifically included in the meta-analysis [30–33].

All six studies examined the cardioprotective efficacy of allopurinol in the setting of coronary artery bypass (CABG) surgery and were published between 1988 and 1995 (Table 1). Of these, four studies included patients undergoing elective CABG [15, 30, 32, 33] and two studies did not specify if participants underwent urgent or non-urgent CABG [29, 31]. Coghlan et al. (1994) exclusively recruited patients undergoing their first CABG [31]. Other studies did not specify if participants had previously undergone CABG surgery [15, 29, 30, 32, 33]. Sample sizes across the included studies ranged from 14 to 90 patients (mean = 38). In the assessed studies, the definition of MI varied (Additional file 1: Table S1) [15, 29–33].

**Table 1:** Study characteristics

Study	n	Country	Allopurinol/Control	Operative Details/Mean CPB Time (min)	Treatment Protocol Allopurinol	Treatment Protocol Control	Emergency CABG (%)	Redo CABG	Mean Graft Number (n)	Mean AXC Time (min)
Emerit et al. (1988)	14	France	7/7	CPB, cardioplegic cardiac arrest/ NR	100 mg, in cardioplegic solution, 2× doses intraoperatively; TD: 200 mg	NT	NR	NR	2.3	50.0
Rashid et al. (1991)	90	Sweden	45/45	CPB, cardioplegic cardiac arrest/123.5	300 mg, PO, BD for 2 days pre surgery and 600 mg, PO, 1× dose morning of surgery and 300 mg, PO, BD for 2 days post-surgery; TD: 3000 mg	NT	0	NR	3.6^a^	76.5
Coghlan et al. (1994)	50	England	25/25	CPB, intermittent ischaemic arrest (*n* = 37); CPB, cardioplegic cardiac arrest (*n* = 13)/116.5	300 mg, PO, 1× dose at 2000 h nocte pre surgery and 1× dose 1 h pre surgery; TD: 600 mg	Placebo	NR	0	3.7	44.5
Taggart et al. (1994)	20	England	10/10	CPB, cardioplegic cardiac arrest/66.5	600 mg, PO, 1× dose nocte pre surgery and 1× dose at 0600 h morning of surgery; TD: 1200 mg	NT	0	NR	2.9	33.5
Castelli et al. (1995)	33	Italy	18/15	CPB, cardioplegic cardiac arrest/NR	200 mg, IV, 1× dose 1 h before Surgery; TD: 200 mg	NT	0	NR	3.0	65.5
Gimpel et al. (1995)	22	The Netherlands	8/14	CPB, cardioplegic cardiac arrest/126.9	200 mg, IV, 1× dose during anaesthetic induction and 100 mg IV, 1× dose hourly in CPD; TD: CPB duration dependant	NT	0	NR	3.4	79.3

*CPB* cardiopulmonary bypass, *NR* not reported, *PO* per os, *BD* twice per day, *TD* total dose, *IV* intravenous, *NT* no treatment, *CABG* coronary artery bypass graft, *AXC* aortic cross-clamp, *NR* not reported. The number of decimal places reported in the included studies varied. Data has been rounded up to one decimal point where appropriate

The number of decimal places reported in the included studies varied. Data has been reported consistently where possible in the table by rounding up of numbers were appropriate

^a^indicates average number of distal anastomoses

All included studies utilised on-pump CABG; however, methods of myocardial protection varied. Cardioplegic cardiac arrest was used in five studies [29–33], including a small subset of patients (*n* = 13) studied by Coghlan et al. (1994). Cardioplegia was most commonly achieved with St. Thomas’ II cardioplegic solution. In contrast, intermittent ischaemic arrest was applied in two studies [15, 31], including a large subset of patients (*n* = 37) studied by Coghlan et al. (1994). Five studies employed hypothermia in the range of 27–34 degrees Celsius [15, 30–33]. There was significant variation in the reporting of baseline variables, and there was minimal reporting of relevant risk factors (Table 2). In total, 229 patients were studied. With respect to reported patient characteristics, including age, sex and preoperative ejection fraction, three studies explicitly stated that there were no statistically significant differences between the treatment groups [15, 31, 33].

**Table 2:** Participants characteristics

Study	n	Mean Age	Male (%)	Mean EF (%)	Mean NYHA Class	Triple Vessel Disease (%)	≥1 anti-anginal medication (%)	Previous MI (%)	DM (%)	Renal Disease (%)	Prior use of Allopurinol
Emerit et al. (1988)	14	NR	NR	NR	NR	NR	NR	NR	NR	NR	NR
Rashid et al. (1991)	90	62	76	> 50^a^	2.3	NR	NR	NR	NR^e^	NR^e^	NR
Coghlan et al. (1994)	50	58	84	63	NR^b^	92	NR^d^	34	14	NR	NR
Taggart et al. (1994)	20	60	100	53	NR	NR	NR	NR	NR	0	0
Castelli et al. (1995)	33	61	94	64	3	NR	NR	79	NR	NR	NR
Gimpel et al. (1995)	22	59	77	NR	NR^c^	NR	100	50	NR	NR	NR

*EF* ejection fraction, *NYHA* New York Heart Association, *MI* myocardial infarction, *DM* diabetes mellitus; NR, not reported

The number of decimal places reported in the included studies varied. Data has been rounded up to whole where appropriate

^a^Ejection fraction > 50% in 71% of the allopurinol group and 67% of the control group

^b^Mean NYHA class not specified. 80% of (n) with NYHA class 3 or 4

^c^Mean NYHA class not specified. 100% of (n) with NYHA class 3 or 4

^d^Reports nitrate use in 10%, beta-blocker use in 46% and calcium channel blocker use in 44%

^e^Prevalence of ‘other organ disease/dysfunction’ reported to be zero. It was unclear whether this included previous cerebrovascular disease, DM, renal disease and/or PAD

With regards to operative factors, all included studies reported the average duration of ischaemia or aortic cross-clamp time (mean = 62.69 min) [15, 29–33]. However, average extra-corporeal perfusion time was only described in four studies (mean = 115.53 min) [15, 30, 31, 33]. Five studies presented data on the average number of grafts placed during CABG (mean = 3.22) [15, 29, 31–33]. In contrast, Rashid et al. (1991) reported the average number of peripheral anastomoses [30]. Notably, no study commented on the average duration of surgery or target-artery quality within their study populations, which are factors known to be associated with the risk of perioperative MI in patients undergoing CABG [34]. With respect to reported intraoperative factors, including number of grafts, and bypass and cross clamp times, only three studies explicitly stated that the allopurinol and control cohorts were statistically similar [15, 31, 33].

Table 3 details MI diagnostic criterion and follow-up periods for all included studies. All included studies focused on the diagnosis of MI in the perioperative setting. Only Coghlan et al. (1994), Taggart et al. (1994) and Gimpel et al. (1995) specified the exact duration of follow up [15, 31, 33]. All six studies described the use of specific criterion for the diagnosis of MI however no study reported on the sensitivity and specificity of their respective criterion [15, 29–33]. Five studies described the combined use of electrocardiogram findings and enzyme studies [15, 29, 30, 32, 33]. In contrast, Coghlan et al. (1994) relied solely upon electrocardiogram criterion for diagnosis [15, 31]. Only Taggart et al. (1994) reported the use of cardiac troponins (cTnT) in conjunction with the creatine kinase-myocardial b isoenzyme for the diagnosis of MI [15]. Nevertheless, the diagnostic criteria reported in all included studies may be considered to be valid for the diagnosis of MI at the time the trials were published [15, 29–33]. Collating results from all included studies [15, 29–33], MI was reported in 16 (6.99%) study participants. Within the pooled allopurinol cohort (*n* = 113), MI was reported in 2 (1.77%) patients. In comparison, MI was diagnosed in 14 (12.07%) patients of the pooled control cohort (*n* = 116). Only one study recorded a statistically significant reduction in the incidence of perioperative MI among patients administered allopurinol (*p* < 0.01) [30].

**Table 3:** Incidence of MI in the included studies by treatment allocation

Study	ECG	Enzyme Studies	Imaging/Angiography/Autopsy	Follow Up	Incidence of MI: Total	Incidence of MI: Allopurinol	Incidence of MI: Control	*P* value
Emerit et al. (1988)	Y^a^	Y^a^	N	NR	0 (0%)	0 (0%)	0 (0%)	NR
Rashid et al. (1991)^d^	Gross changes^a^	AST, ALT > 2.5 μkat/L^b^ LDH > 8 μkat /L CK-MB > 1.7 μkat t/L	N	NR	8 (8.9%)	0 (0%)	8 (17.8%)	< 0.01
Coghlan et al. (1994)	New Q waves	N	N	During CABG only	3 (6.0%)	1 (4.0%)	2 (8.0%)	NR
Taggart et al. (1994)	New persistent Q waves (> 0.04 ms) or loss of > 25% of R waves in ≥2 leads^c^	N	N	72 h post-CABG	0 (0%)	0 (0%)	0 (0%)	NS
Castelli et al. (1995)	Y	Plasma CPK-MB > 50 IU/ml	N	NR	3 (9.1%)	1 (5.6%)	2 (13.3%)	NS
Gimpel et al. (1995)	New Q waves	Plasma CK-MB > 15 U/L	N	10 days post-CABG	2 (9.1%)	0 (0%)	2 (14.3%)	NS

*ECG* electrocardiogram, *Y* Yes, *N* No, *NR* not reported, *AST* aspartate transaminase, *ALT* alanine transaminase, *LDH* lactate dehydrogenase, *CK-MB* creatine kinase-MB, *CABG* coronary artery bypass graft, *CPK-MB* creatine phosphokinase-MB, *NS* not significant, *NR* not reported

^a^Nil further details provided

^b^Especially if AST elevated in the presence of normal ALT

^c^In isolation, minor ST-T wave changes or changes in conduction were not considered diagnostic of perioperative MI

^d^Diagnostic criterion also considered the general condition of patient and signs of post-operative infarction (not specified)

As methods of randomisation and allocation concealment were frequently unreported, the risks of selection bias were largely unclear (Table 4). Three studies described the use of coin tossing, random number tables and computer programmes to achieve randomisation [15, 30, 31]. Additionally, Coghlan et al. (1994) detailed the use of identical, sequentially numbered drug containers to conceal allocation [31]. Hence, these studies were deemed to carry low risks of selection bias. Only Coghlan et al. (1994) employed a double-blinded, placebo-controlled study design wherein the blinding code remained unbroken until all outcome assessments were completed [31]. In Rashid et al. (1991), there was incomplete blinding of clinical staff and it was unclear if all outcome assessors were blinded to the treatment groups [30]. Excluding the above mentioned studies, blinding of clinical staff and outcome assessors were not discussed in any other study [15, 29, 32, 33]. Patient blinding was only reported in the trial authored by Coghlan et al. (1994) [15, 29, 30, 32, 33]. Therefore, only the study reported by Coghlan et al. (1994) could be determined to carry a low risk of performance and detection bias [31].

**Table 4:** Risk of Bias

Study	Random Sequence Generation	Allocation Concealment	Blinding of participants and personnel	Blinding of outcome assessments	Incomplete outcome data	Selective reporting	Other Bias
Emerit et al. (1988)	U	U	U	U	U	H	U
Rashid et al. (1991)	L	U	U	U	L	U	U
Coghlan et al. (1994)	L	L	L	L	L	H	H
Taggart et al. (1994)	L	U	U	U	L	U	U
Castelli et al. (1995)	U	U	U	U	L	H	H
Gimpel et al. (1995)	U	U	U	U	L	U	U

*U* unclear, *L* low, *H* high

Five of the six included studies reported complete follow-up of all randomised patients and intention-to-treat analyses [15, 30–33]. Thereby, the risk of attrition bias was generally low. Bias also arose from selective reporting. For example, Castelli et al. (1995) did not report on pre-defined haemodynamic outcomes such as peripheral blood pressure after CABG. Similarly, Emerit et al. (1988), and Coghlan et al. (1994) did not present data on predefined clinical outcomes such as the incidence of postoperative arrhythmias, haemorrhage and reoperation [29, 31]. Two included studies also had other potential sources of bias [31, 32]. In particular, Coghlan et al. (1994) and Castelli et al. (1995) measured multiple primary outcomes from small, non-randomly selected, patient sub-groups [31, 32].

**Fig. 2:**
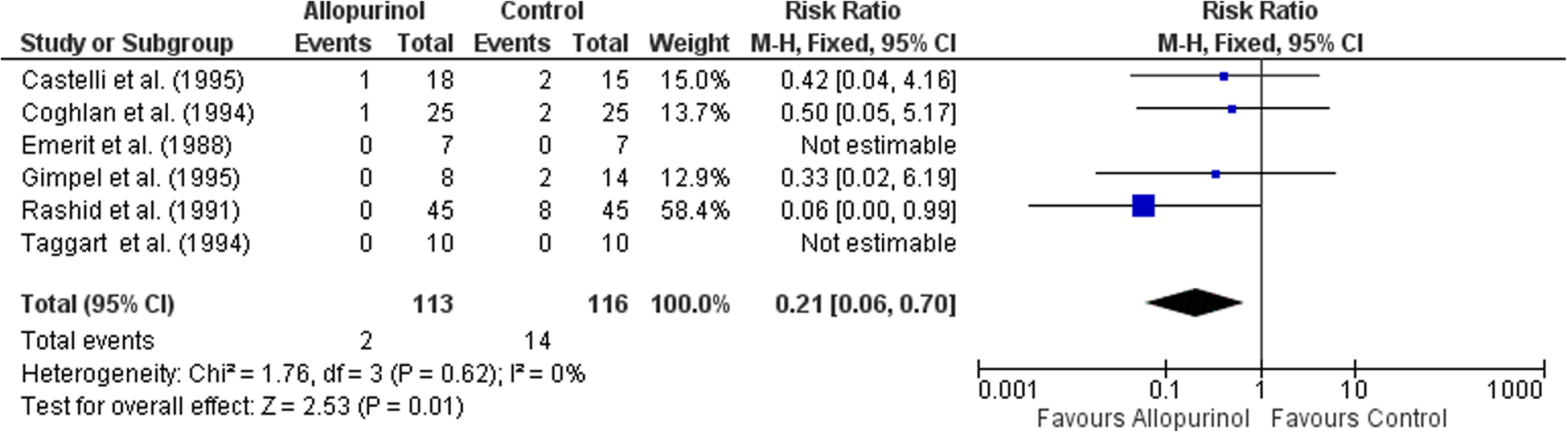
Forest plot illustrating risk ratios for MI in participants on Allopurinol treatment. Square boxes indicate risk ratios in the primary studies for the risk of MI. The size of the box reflects the statistical weight of the study. Horizontal lines indicate the 95% confidence intervals (CI). The diamond represents the overall risk ratio and 95% CI (RR 0.21, 95% CI 0.06–0.70, *p* = 0.01), calculated using a fixed effects meta-analysis

Combining all included studies (*n* = 6), an I^2^ statistic of 0% was obtained [15, 29–33]. Based on guidelines from the Cochrane Handbook 5.1.0, this value represents non-significant heterogeneity [25]. Using a fixed-effects model, allopurinol was found to significantly reduce the incidence of MI (RR 0.21; 95% CI: 0.06–0.70; *p* = 0.01) (Fig. 2). In the leave-one-out sensitivity analysis (Additional file 1: Table S2), the treatment effect became non-significant with the removal of the trial reported by Rashid et al. (1991). Due to the small number of studies included within this review (i.e *N* < 10), the potential influence of publication bias could not be assessed reliably.

## Discussion

In this systematic review and meta-analysis, allopurinol was found to significantly decrease the incidence of perioperative MI in patients undergoing CABG. The treatment effect of allopurinol was found to be greatest in the trial conducted by Rashid et al. (RR 0.06 95% CI: 0.00–0.99, *p* = 0.05), wherein patients had the largest and longest exposure to allopurinol i.e. 3000 mg, over five days. Furthermore, sensitivity analyses suggested that removal of the latter trial led to the treatment effect becoming non-significant.

In the context of previous research within this field, the findings of this review are not isolated. Among patients undergoing CABG and cardiac valve replacement surgery, Tabayashi et al. (1991) found that high-dose allopurinol administration significantly decreased ischaemic markers including aspartate transaminase, creatine kinase and lactate dehydrogenase in the postoperative period [35]. Similarly, in a double-blind, placebo-controlled study, Movahed et al. (1996) reported that allopurinol could decrease lipid peroxidation as evidenced by reduced malondialdehyde levels and attenuate ischaemic injury as measured by creatine kinase-myocardial b isoenzyme levels [36].

There are a number of proposed mechanisms which could explain the cardioprotective effects of allopurinol [37]. Firstly by lowering circulating concentrations of uric acid. Elevated serum uric acid levels have been associated with increased levels of proinflammatory cytokines, increased oxidation of low-density lipoprotein (LDL) and release of platelet constituents and subsequent pro-thrombotic effects [6, 38]. Perhaps most striking is uric acids putative role in hypertension via inhibiting nitric oxide production and promoting the effects of angiotensin II [5, 39, 40]. In contrast, it has also been suggested that elevated serum uric acid levels following ischaemic insult denotes a physiological and protective response to oxidative stress [41, 42]. Secondly, allopurinol is thought to inhibit xanthine oxidase mediated generation of reactive oxygen species, limiting endothelial dysfunction and atherosclerosis plaque instability [2, 3, 7–12]. In response to xanthine oxidase inhibition, serum markers of oxidative stress have been shown to reduce in subjects with a range of cardiovascular diseases [2]*.* Finally, it has also been suggested that allopurinol inhibits purine catabolism thereby increasing local tissue availability of adenosine triphosphate and oxygen [8, 13, 14]. In an animal model study by Ekuland et al. (1999), allopurinol was reported to posses ionotropic properties while simultaneously reducing energy requirements and myocardial oxygen consumption [14].

Interestingly, randomised controlled trials investigating the cardioprotective efficacy of allopurinol have largely focussed on operative settings such as CABG surgery. Therefore, this review could not explore if long-term allopurinol exposure decreased the risk of MI in other settings. This represents a major gap in our knowledge. Additionally, all trials included in this review were small-scale studies published some years ago. Included studies were limited to the English language and it is possible that this may have introduced bias in our analysis. As Consolidated Standards of Reporting Trials was not well established at the time, there were significant discrepancies in the reporting of randomisation, blinding and outcome assessment. Moreover, surgical techniques and MI diagnostic algorithms have changed substantially since these studies were published and as such, it is unclear if and to what extent, these results are applicable to current clinical practice. In light of these insights, further research is required to better understand the potential benefits of allopurinol in MI prevention. Large trials are needed to definitively corroborate the cardioprotective efficacy of allopurinol, over longer follow up durations. Additional studies are also required to determine whether the cardiovascular benefits of allopurinol are dose dependant as previously suggested [43, 44]. The ALL-HEART [45] study is a multi-centre randomised controlled trial which is investigating the impact of high-dose allopurinol therapy (600 mg daily) on major cardiovascular outcomes (non-fatal MI, non-fatal stroke, or cardiovascular death) in patients with ischaemic heart disease with an expected average follow-up of 4 years. It is hoped that evidence from such trials will help confirm the potential cardiovascular benefits of allopurinol prescription.

## Conclusion

Based on limited evidence from small randomised trials published over twenty years ago, allopurinol appears to decrease the incidence of perioperative MI following CABG. However, further research is required to confirm and characterise the relationship between allopurinol and cardiovascular outcomes including MI.

## Additional file

Additional file 1: **Table S1.** Diagnostic criteria for MI. **Table S2.** Sensitivity Analyses using the leave-one-out approach. (DOCX 18 kb)

## Abbreviations

CABG: Coronary artery bypass
CI: Confidence intervals
LDL: Low-density lipoprotein
MI: Myocardial infarction
RR: Risk ratio
XOI: Xanthine oxidase inhibitor
