# Supplementary material for: A meta-analysis of the efficacy of allopurinol in reducing the incidence of myocardial infarction following coronary artery bypass grafting
Source: BMC Cardiovasc Disord. 2018 Jul 11;18:143. doi: 10.1186/s12872-018-0881-6 (PMC6042232; doi:10.1186/s12872-018-0881-6)
Supplement: Supplementary file 1 — Table S1. Diagnostic criteria for MI. Table S2. Sensitivity Analyses using the leave-one-out approach. (DOCX 18 kb) [file 12872_2018_881_MOESM1_ESM.docx]

**Table S1:** Diagnostic criteria for MI.

| **Study** | **Diagnostic criteria utilized for MI*** |
| --- | --- |
| Castelli *et al.* (1995) | Based on ECG recording and plasma >50 IC/ml CPK-MB activity |
| Rashid *et al.* (1991) | Based on signs, pathological increase of transaminases and enzymes; aspartate transaminase and alanine transaminase (especially if an increase in only aspartate transaminase not alanine transaminase; the cut off level is greater than 2.5 pkat/L, and creatinine-kinase-MB is greater than 8 pkat/L, and creatine kinase-MB is greater than 1.7pkat/L. |
| Coghlan *et al.* (1994) | NR |
| Taggart *et al.* (1994) | Serial electrocardiograms were performed at one, six, 12, 24 and 72 hours after surgery. A new persistent Q wave (>0.04ms) or loss of >25% of R waves in at least two leads were considered indicative of perioperative infarction. |
| Gimpel *et al.* (1995) | ECG examined for new Q waves during the first two postoperative days and one t day 10. Blood samples for CK-MB isoenzyme measurements were taken 2,4,8,12 and 20 hours post-operatively. |
| Emerit *et al.* (1988) | NR |

NR; Not reported.

**Table S2:** Sensitivity Analyses using the leave-one-out approach.

| **Study removed** | **RR (95% CI)** | ***p*-value** |
| --- | --- | --- |
| Castelli *et al.* (1995) | 0.17 (0.04, 0.73) | *p* = 0.02 |
| Coghlan *et al.* (1994) | 0.16 (0.04, 0.70) | *p* = 0.01 |
| Gimpel *et al.* (1995) | 0.19 (0.05, 0.73) | *p* = 0.02 |
| Rashid *et al.* (1991) | 0.42 (0.10, 1.75) | *p* = 0.23 |
| Emerit *et al.* (1988) | 0.21 (0.06, 0.70) | *p=* 0.01 |
| Taggart *et al.* (1994) | 0.21 (0.06, 0.70) | *p=* 0.01 |

RR, risk ratio. Indicates the pooled results with the corresponding study excluded from the analysis.
